# Recombinant adeno-associated virus expressing a p53-derived apoptotic peptide (37AA) inhibits HCC cells growth *in vitro* and *in vivo*

**DOI:** 10.18632/oncotarget.15160

**Published:** 2017-02-06

**Authors:** Hongyong Zhang, Yufeng Wang, Yanxia Bai, Yuan Shao, Jigang Bai, Zhenhua Ma, Qingguang Liu, Shengli Wu

**Affiliations:** ^1^ Department of Hepatobiliary Surgery, The First Affiliated Hospital of Xi’an Jiaotong University, Xi’an, Shaanxi 710061, P.R. China; ^2^ Department of Otorhinolaryngology, The First Affiliated Hospital of Xi’an Jiaotong University, Xi’an, Shaanxi 710061, P.R. China

**Keywords:** recombinant adeno-associated virus, P53, P73, HCC, 37AA

## Abstract

Recent studies have confirmed that a p53-derived apoptotic peptide (37AA) could act as a tumor suppressor inducing apoptosis in multiple tumor cells through derepressing p73. However, the tumor suppressive effects of recombinant adeno-associated virus (rAAV) expressing 37AA on HCC cells are still unknown. In this study, we successfully constructed a recombinant rAAV expressing 37AA. *In vitro* and *in vivo* assays showed that transfection of NT4-37AA/rAAV in HCC cells strongly suppressed cell proliferation, induced apoptosis, and up-regulated the cellular expression of p73. NT4-37AA/rAAV transfection markedly slowed Huh-7 xenografted tumor growth in murine. Pretreatment of HCC cells with p73 siRNA abrogated these effects of NT4-37AA/rAAV. Furthermore, we found that expression of p73 was upregulated and the formation of P73/iASSP complex was prevented when 37AA was introduced into HCC cells. Taken together, these results indicate that introduction of 37AA into HCC cells with a rAAV vector may lead to the development of broadly applicable agents for the treatment of HCC, and the mechanism may, at least in part, be associated with the upregulation of p73 expression and reduced level of P73/iASSP complex.

## INTRODUCTION

Hepatocellular carcinoma (HCC) is one of the most deadly cancers in humans [1]. Despite considerable therapeutic progress, patients with HCC still face a high incidence of postoperative recurrence and metastases, even when treatments have been considered potentially curative [2]. Sorafenib is the only and standard systematic chemotherapeutic agent for unresectable HCC currently. However, its clinical benefits remain modest with an average overall survival of only 10.7 months for advanced HCC patients [3]. In general, the overall prognosis of HCC is still unsatisfactory.

Recent advances in the molecular pathogenesis of HCC revealed that complex genetic and epigenetic alterations, chromosomal aberrations, mutations, and altered molecular pathways lead to the development of HCC [4, 5]. Analyses of these aberrant alterations involved in the development of HCC could help us to identify potential diagnostic markers and new molecular targets of HCC. Various targeted therapies are currently being explored for combating HCC, and accumulating evidence suggests that combination therapy targeting different pathways, such as angiogenesis, signal transduction and epigenetic dysregulation of tumors, will potentiate anti-HCC effects and provide perspectives on future clinical trials in HCC [6, 7].

Mutation of the p53 tumor suppressor gene is a common genetic change in HCC, present in 30% of cases [8]. Functionally, p53 acts as a tumor suppressor through several mechanisms including activating DNA repair proteins, inducing cell cycle arrest, and initiating apoptosis [9, 10] and when mutated, its tumor suppressor ability would be impaired or even eliminated, which makes p53 pathway an ideal target for therapeutic intervention in multiple tumor types including HCC [11, 12]. However, the technical bottleneck of restoring normal function to a mutant tumor suppressor gene or inactivating it makes the p53 protein-based HCC treatment theoretically easy, practically rather difficult.

P73 is a member of the p53 family which shares many functional characteristics with p53 and can induce cell death in multiple cell types [13–16]. In contrast to p53, p73 is rarely mutated in human cancer [17, 18], which provided the possibility to fight against tumor via activating p73 in p53 mutated or p53 null tumors. Recently, Bell et al. [19] reported a p53-derived apoptotic peptide (37AA), which composed of only 37 amino acids from conserved box II (residues 118–142) and conserved box III (residues 171–181). Although unable to directly cause p53 target gene activation, 37AA retains the ability to bind the inhibitor of apoptosis stimulating protein of p53 (iASPP), regardless the status of P53. By binding iASPP, 37AA could dissociate iASPP and p73 to result in the derepression of endogenous p73, which subsequently causes p73 activation and p73-dependent cell death and tumor regression in the absence or presence of p53 mutation.

In this study, we successfully constructed a recombinant rAAV expressing 37AA and selected p53 mutated cell line Huh-7, and p53 null cell line Hep3B to examine the tumor suppressive effects of 37AA *in vitro*. Besides, a Huh-7 xenografted tumor in mouse was used to evaluate its tumor suppressive effects *in vivo* and the possible mechanisms were also investigated.

## RESULTS

### Titration of NT4-37AA/rAAV

After the construction of NT4-37AA/rAAV, the titer was measured by spot hybridization method. The result showed its titer reached a maximum of 2 × 10^13^ pfu/L.

### Knockdown of p73 in HCC cells

**Figure 1:**
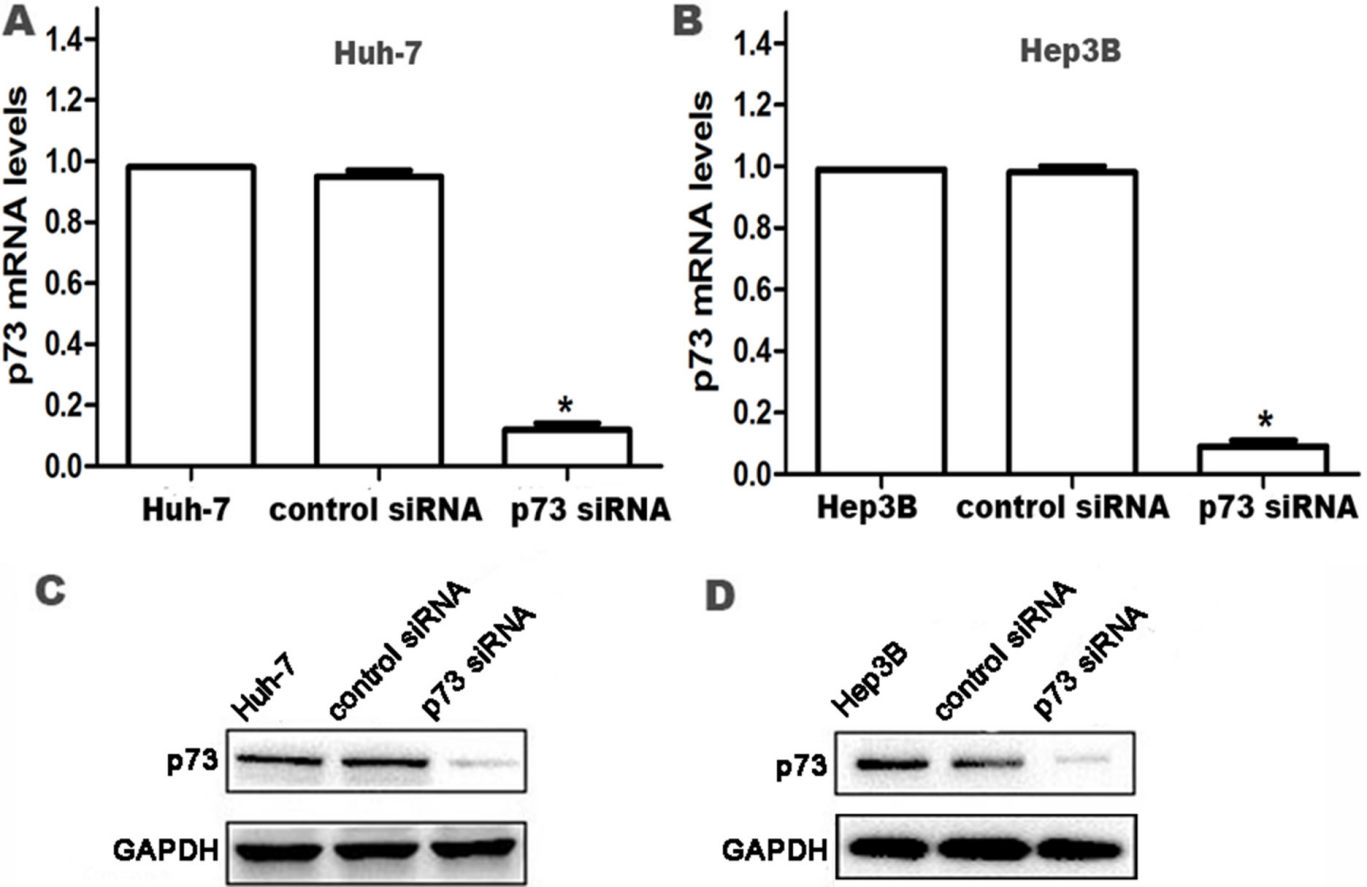
Knockdown of p73 expression in HCC cells at 24 h after siRNA transfection (**A**, **B**) p73 mRNA levels were determined by real time RT-PCR. Relative fold induction for p73 mRNA (means ± SD) in the control siRNA- and p73 siRNA-transfected Huh-7 and Hep3B cells was presented relative to the expression in the parental Huh-7 and Hep3B cells (*P < 0.05 compared with parental Huh-7 and Hep3B cells and control siRNA-transfected Huh-7 and Hep3B cells, respectively). (**C**, **D**) Western blot analysis for p73 protein expression in parental Huh-7 and Hep3B cells, control siRNA- and p73 siRNA-transfected Huh-7 and Hep3B cells, respectively. GAPDH was used as an internal control.

The expression of p73 was examined by real-time PCR and western blot to validate the silencing efficiency of the target gene after RNA interference. Stable p73 siRNA-transfected Huh-7 and Hep3B cells (p73-siRNA group) and control siRNA-transfected cells (control siRNA group) were established as described above. Compared to parental Huh-7 and Hep3B cells and control siRNA cells, both mRNA and protein expression of p73 were significantly reduced in p73-siRNA cells at 24 h after siRNA transfection (all *P* < 0.05; Figure 1), which persisted for at least 96 h (data not shown).

### NT4-37AA/rAAV increases the expression of p73 protein in HCC cells

**Figure 2:**
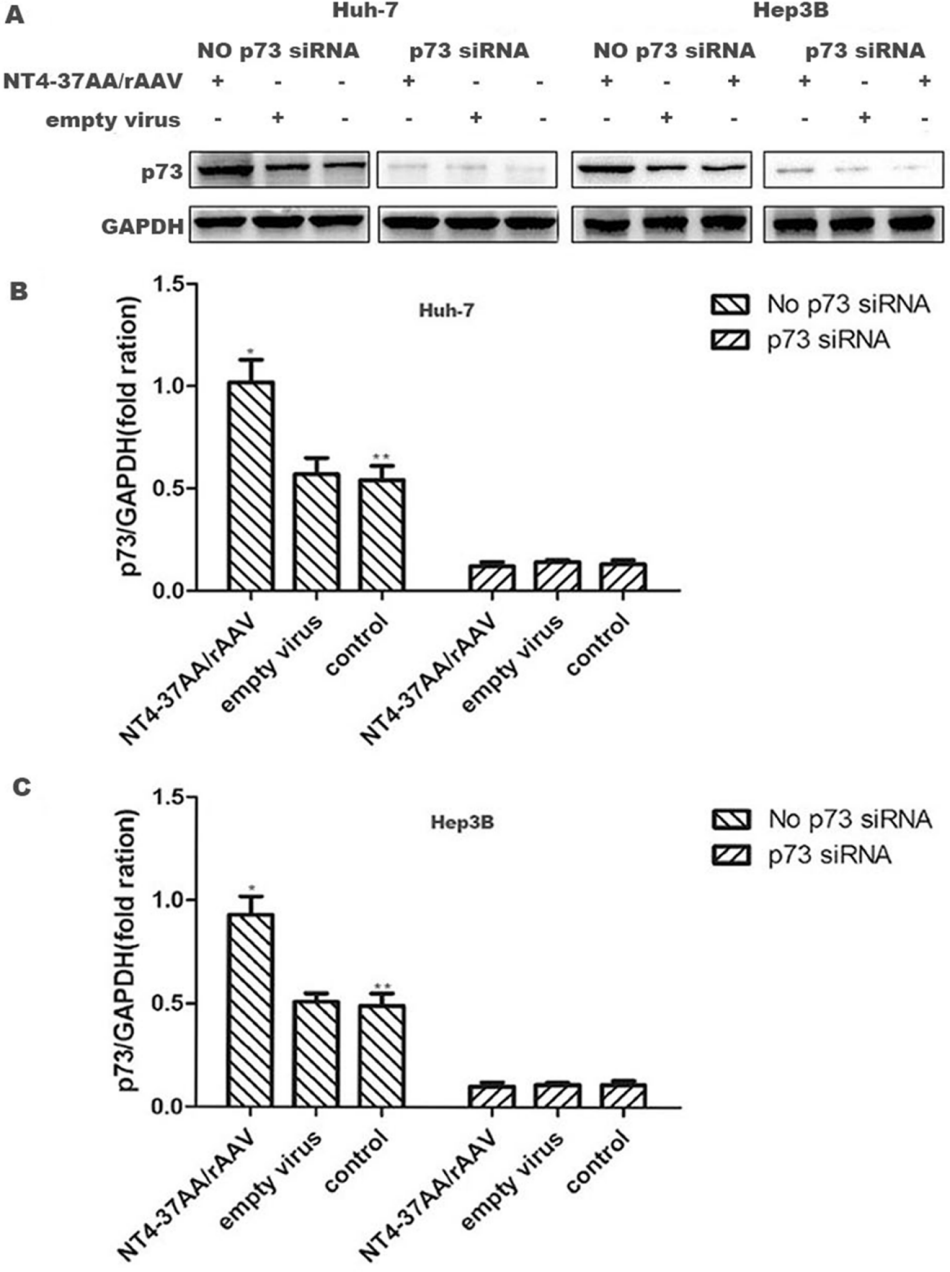
Transfection of NT4-37AA/rAAV increased the expression of p73 protein in HCC cells (**A**) The Huh-7 and Hep3B cells received or not received p73 siRNA pretreatment were treated with NT4-37AA/rAAV or empty virus or nothing, respectively, for 72 hours. The protein expression levels of p73 were determined by western blot. (**B**, **C**) The fold changes in intensity normalized by GAPDH were shown by densitometric analysis (*P < 0.05 compared with the other two groups without p73 siRNA pretreatment, respectively; **P < 0.05 compared with the three groups with p73 siRNA pretreatment, respectively).

For the Huh-7 and Hep3B cells not received p73 siRNA pretreatment, the expression of p73 protein in NT4-37AA/rAAV infected cells was significantly higher than that in the other two groups (both *P* < 0.05, Figure 2).

However, the up-regulating effect of NT4-37AA/rAAV on p73 protein in Huh-7 and Hep3B cells was abrogated by the pretreatment of Huh-7 and Hep3B cells with p73 siRNA. For the three groups of Huh-7 and Hep3B cells received p73 siRNA pretreatment, the p73 expression levels were similar and all significantly reduced compared to that in control cells not received p73 siRNA pretreatment (all *P* < 0.05, Figure 2).

### The inhibitory effect of NT4-37AA/rAAV on HCC cells proliferation

**Figure 3:**
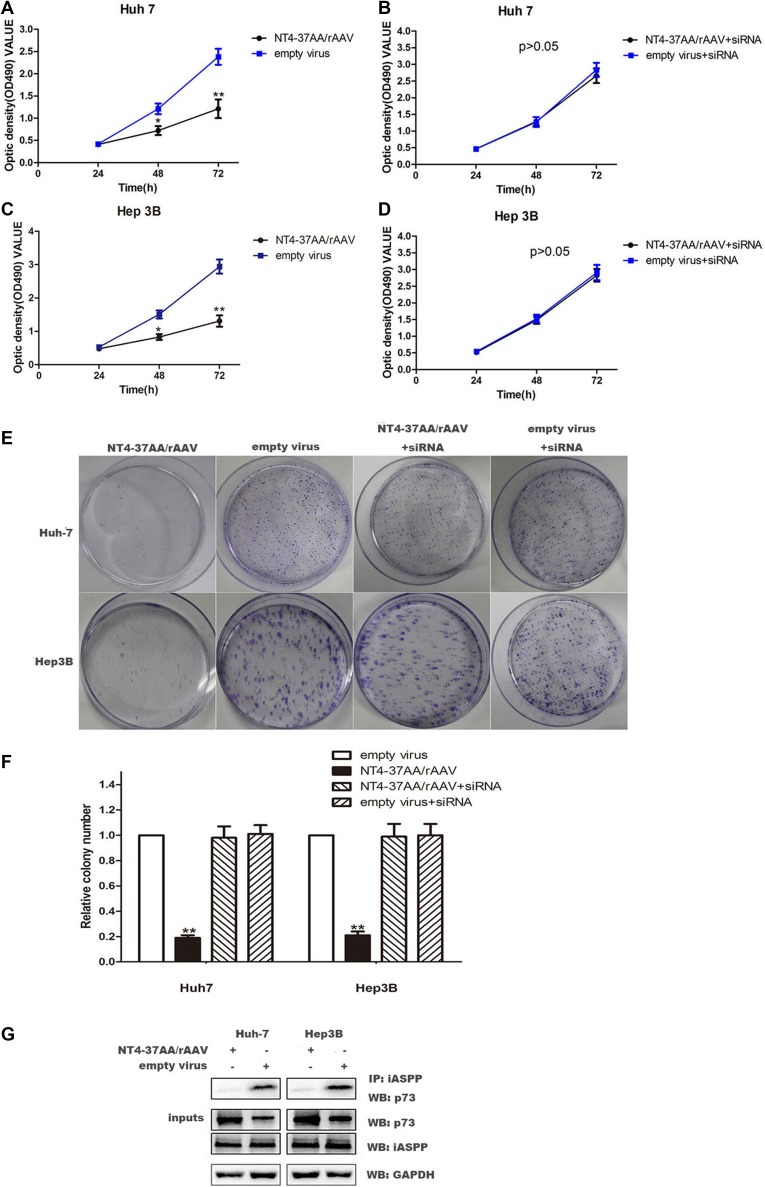
Transfection of NT4-37AA/rAAV reduced cell proliferation in HCC cells (**A**, **B**, **C**, **D**) Cell proliferation was analyzed using the MTT assay. Cells were monitored for 72 h and the average OD490 (± SD) for each cell line is shown (*P < 0.05, **P < 0.01 compared with the empty virus group). (**E**, **F**) The effects of NT4-37AA/rAAV and p73 siRNA on long-term cellular survival were determined by assessing the clonogenic capacity of the cells (**P < 0.01 compared with the other groups). (**G**) P73 and iASPP coimmunoprecipitate with each other. NT4-37AA/rAVV was transfected into Huh-7 cells and Hep3B cells. iASPP was immunoprecipitated with anti-iASPP antibody. Western blot was performed to detect the specific proteins labeled on the right side of each panel.

In order to explore the inhibitory effect of NT4-37AA/rAAV on HCC cells proliferation, we carried out MTT and colony formation assay to explore the changes of HCC proliferation. For the Huh-7 and Hep3B cells not received p73 siRNA pretreatment, the proliferation of cells treated with recombinant virus was significantly restrained compared to the empty virus group (all *P* < 0.05, Figure 3A, 3C, 3E and 3F).

However, the inhibitory effect of NT4-37AA/rAAV on Huh-7 and Hep3B cells proliferation was abrogated by the pretreatment of Huh-7 and Hep3B cells with p73 siRNA. For the Huh-7 and Hep3B cells received p73 siRNA pretreatment, the OD values and colony number were similar to the empty virus group (Figure 3B, 3D, 3E and 3F).

To further explore the mechannism of how NT4-37AA/rAAV inhibits HCC cell proliferation, we conducted CO-IP. The results showed that NT4-37AA/rAAV could reduce the formation of the P73 / iASSP complex (Figure 3G).

### NT4-37AA/rAAV increased the apoptosis of HCC cells

**Figure 4:**
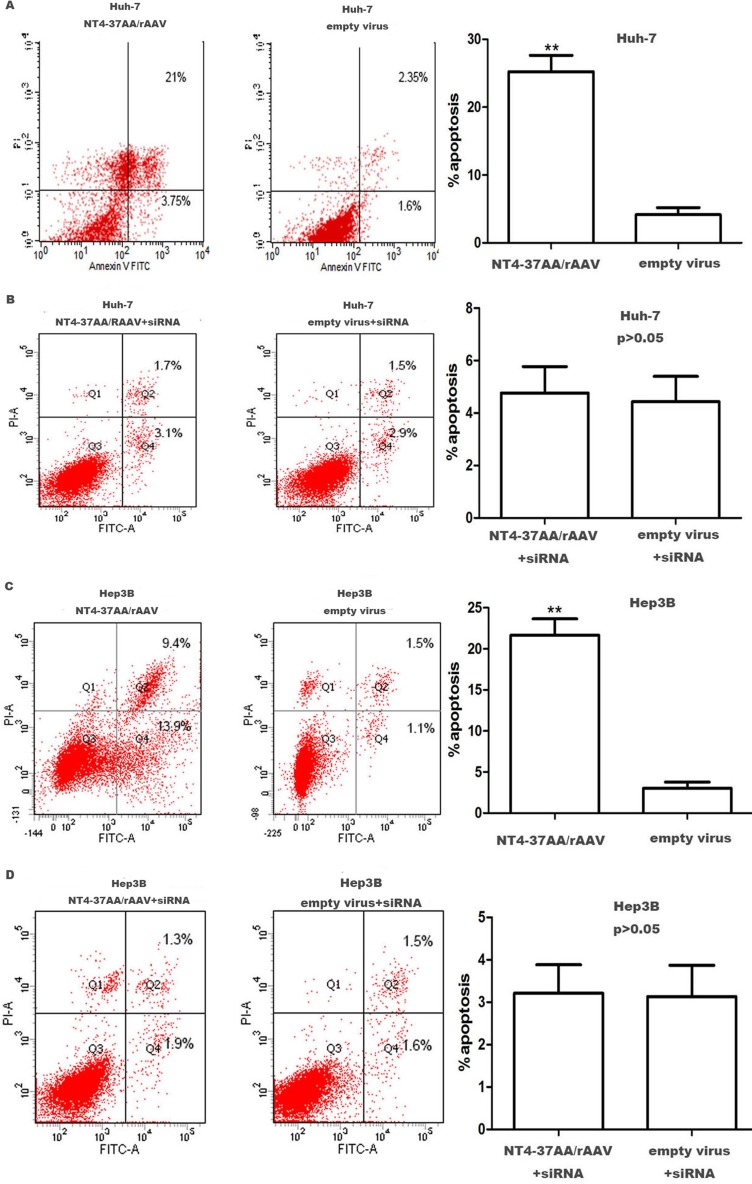
Transfection of NT4-37AA/rAAV increased the apoptosis of HCC cells Quantification of the apoptotic cell population was determined by flow cytometry. (**A**, **C**) Both Huh7 and Hep3B cells with overexpressed NT4-37AA/rAAV were composed of a larger subset of apoptotic cells compared with the control cells. (**B**, **D**) There was no significant difference when cotransfected with p73 siRNA (**P < 0.01 compared with the empty virus group).

For the Huh-7 and Hep3B cells not received p73 siRNA pretreatment, flow cytometry analysis showed that the recombinant virus could increase the apoptosis rate of the cells. After being infected for 72 h, the apoptosis rate of Huh-7 and Hep3B cells in recombinant virus group was much higher than that in the empty virus group (Figure 4).

However, the induction of apoptosis by NT4-37AA/rAAV in Huh-7 and Hep3B cells was abrogated by the pretreatment with p73 siRNA. For the Huh-7 and Hep3B cells receiving p73 siRNA pretreatment, the apoptotic rates were similar and had no significant difference (Figure 4).

### NT4-37AA/rAAV suppressed Huh-7 xenograft tumor growth in nude mice

**Figure 5:**
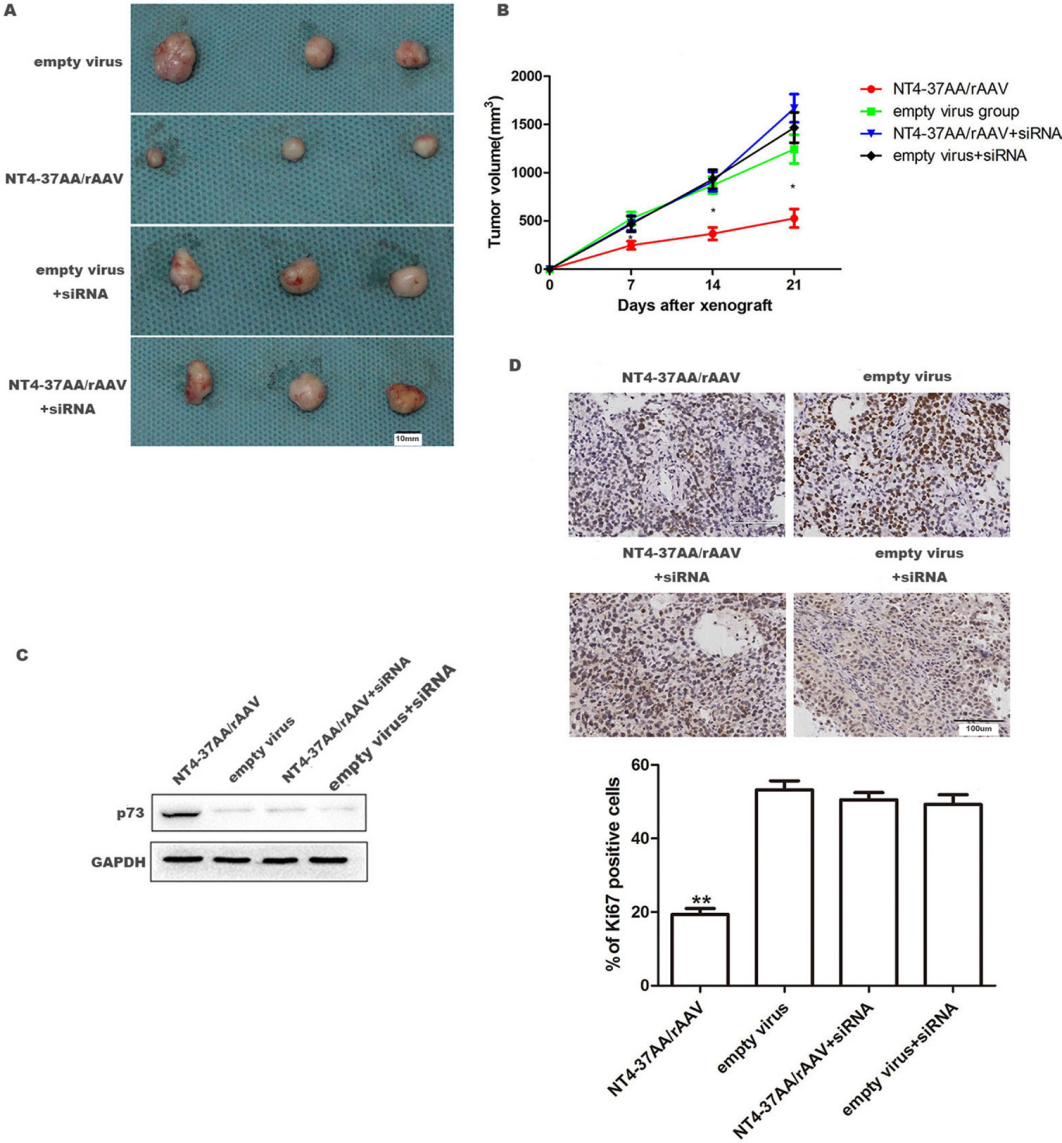
NT4-37AA/rAAV suppressed the growth of Huh-7 xenografts in nude mice 1 × 10^6^ Huh-7 cells received or not received p73 siRNA pretreatment were treated with NT4-37AA/rAAV or empty virus, respectively, and then subcutaneously injected into the dorsal flanks of nude mice. Tumor growth was monitored for 3 weeks. (**A**) Representative pictures of HCC xenografts. Scale bar: 10 mm. (**B**) Tumor volumes (*P < 0.05 compared with other groups). (**C**) Relative p73 expression in HCC xenografts. (**D**) Tumor nodules were subjected to immunohistochemical staining for Ki-67 and quantitative analysis. Operative region under a magnification of ×400. Scale bar: 100 um. Values are depicted as the mean relative level of Ki-67 (**P < 0.01 compared with other groups).

To evaluate the anti-tumor effect of NT4-37AA/rAAV *in vivo*, a HCC xenograft model was successfully established after inoculating Huh-7 cells in the dorsal flanks of nude mice. For the Huh-7 cells not received p73 siRNA pretreatment, NT4-37AA/rAAV substantially attenuated the growth of xenograft tumors as compared to vehicle control at 14 and 21 days post-inoculation, respectively (Figure 5A, 5B).

However, the suppressive effect of NT4-37AA/rAAV on HCC xenograft tumor was abrogated by the pretreatment of Huh-7 cells with p73 siRNA. For the two groups of Huh-7 cells received p73 siRNA pretreatment, the tumor volumes were similar and all slightly greater, but no significant difference, than that in control cells not received p73 siRNA pretreatment (Figure 5A, 5B).

Next, we analyzed p73 expression in the xenograft by western blot. Our result showed that p73 was overexpressed in NT4-37AA/rAAV transfected tumor (Figure 5C). We also performed immunohistochemistry for Ki-67 in the xenografted tissues. As expected, NT4-37AA/rAAV overexpression inhibited cellular proliferation *in vivo*, while p73 siRNA pretreatment could abrogate this effect (Figure 5D).

## DISCUSSION

The p53, a tumor suppressor, is a potent inducer of tumor cell apoptosis. And recently, strategies to exploit p53 for cancer therapy have drawn more and more attention from researchers. Over the past decades, a number of tumor-suppressive consequences of p53 activation have been described. In 2003, Gendicine, a recombinant adenovirus engineered to express wild type p53, was licenced for clinical use in China for head and neck malignancies [20]. In April 2007, Advexin, an adenoviral based p53 gene delivery product, entered its phase III clinical trial in United States and demonstrated anticancer activity following adequate expression of p53 in cancer cells [21]. Currently, most p53 based strategies are through direct or indirect regulations on p53 activity to achieve the tumor-suppressive effects.

However, in about 30% of HCC, p53 mutated and lost their normal functions, which limited the application of HCC gene therapy through p53 activation. Besides, some recent researches about the mechanism of tumor-suppressor proteins, such as PML [22] and VHL [23], revealed that the pathogenesis of tumors included not only the lack of some tumor-suppressor proteins and their mutation, but also their abnormal nuclear localization and rapid degradation. Therefore, the antitumor effect via transduction of complete tumor suppressor gene might be insufficient.

P73 is the homologue of the tumor suppressor p53 and akin to p53 in its functionality based on cell-based assays. In response to apoptotic stimuli, p73 transactivates p53-responsive genes and is capable of inducing cell cycle arrest and apoptosis in multiple mammalian cells [24]. Therefore, p73 can function as a substitute for p53-deficient cancer cells. Previous studies have demonstrated that 37AA, a p53-derived apoptotic peptide, could disrupt the interaction between p73 and iASPP by binding the latter, and then resulted in p73-mediated gene activation and p73-dependent cell death [19].

In the present study, we constructed an AAV vector expressing 37AA, NT4-37AA/rAAV, and explored its tumor suppressive effects on HCC cell growth *in vitro* and *in vivo*. Our studies showed that transfection of NT4-37AA/rAAV in HCC cells strongly restrained cell proliferation, induced apoptosis, and markedly slowed Huh-7 xenografted tumor growth in nude mice. Meanwhile, the expression of p73 was up-regulated and the P73/iASSP complex was prevented in recombinant virus transfected HCC cells. However, all of these *in vitro* and *in vivo* effects of NT4-37AA/rAAV could be abrogated by the pretreatment of HCC cells with p73 siRNA, which suggested that the tumor suppressive mechanism of NT4-37AA/rAAV may, at least in part, be associated with the upregulation of p73 expression and reduced formation of the P73/iASSP complex.

In conclusion, our data demonstrated that introduction of 37AA into HCC cells with rAAV vectors could suppress HCC cells growth *in vitro* as well as *in vivo*. The mechanism may, at least in part, be associated with the upregulation of p73 expression and reduced level of P73/iASSP complex. P73 protein-based adenoviral gene therapy for cancer is theoretically effective and feasible, which would lay a foundation for further research on gene therapy for p53 mutant or missing tumors such as HCC. However, the detailed molecular mechanisms underlying the involvement of 37AA in tumor suppression of HCC are still unknown and further studies need to be performed to address these issues.

## MATERIALS AND METHODS

### Construction and titration of NT4-37AA/rAAV

NT4-37AA/rAAV was prepared according to the protocol described previously [25]. Firstly, the primers were designed according to the DNA sequences of human p53 conserved box II and III, with the corresponding human p53 118-142aa cDNA and human p53 118-142aa cDNA fusioned into one open reading frame (ORF). Primers were synthesized by Sangon Biological Engineering Co., Ltd (Shanghai, China) and the sequences were as follows: Forward primer F1: 5′-cttgcacgtactcccctgccctcaacaagatgttttgccaactggcc-3′; Negative primer R1: 5′-cgcctcacaacctcagggcaggtcttgg ccagttggca-3′; Forward primer F2: 5′-cgccggcgtggga cagccaagtctgtgacttgcacgtactcc-3′; Negative primer R2: 5′-cggtaccccgcgctcatggtgggggcagcgcctcacaacctc-3′. The PCR program included pre-denaturation at 95°C for 5 min, 30 amplification cycles each consisting of denaturation at 94°C for 60 sec, 38°C for 60 sec and 72°C for 80 sec, followed by further extension at 72°C for 5 min. Then the product was linked to plasmid pGEM-T Easy (Promega, USA) and transferred into the competent E. coli DH5α cells using CaCl_2_ method. The successful transferred bacterial colony was selected by enzyme identification and was extracted for pGEM-T Easy/NT4-37AA using alkaline lysis method. Then NT4-37AA was obtained and linked to vector plasmid pSSHG-CMV. Finally, NT4-37AA/rAAV was constructed in HEK-293 cell line co-transfected with pSSHG-CMV/NT4-37AA, auxiliary packaging plasmid pAAV/Ad, and adenovirus genome plasmid pFG140 (the latter two from Huaguang biological engineering company, Xi’an, China) by calcium phosphate coprecipitation method and the titer of recombination adenovirus was determined using spot hybridization method.

### Cell culture and p73 siRNA transfection

Huh-7 and Hep3B cells were cultured in RPMI-1640 medium containing 10% fetal bovine serum in an incubator with 5% CO_2_ at 37°C and relative humidity of 95%. For downregulation of endogenous p73 expression, the following siRNA duplex (Invitrogen, USA) was used: p73 siRNA-1: 5′-UCUGCUUGAAGGCACGCUUGCUGGC-3′ and p73 siRNA-2: 5′-AGUACGUGUCCUCGUCUCCAUGCC G-3′. As a negative control, Stealth RNAi^TM^ siRNA negative control hi GC (12935-400, Invitrogen) was used. Cells were transfected with siRNA for 24 h using Lipofectamine RNAiMAX (Invitrogen) according to the manufacturer's instructions. Specific silencing of targeted genes was confirmed by at least 3 independent experiments.

### Real-time PCR

Real-time PCR was used to determine the levels of p73 transcripts in Huh-7 and Hep3B cells transfected with p73 siRNA for 24 h according to a previous study [26]. p73 gene-specific amplification was confirmed by PCR with specific primers (sense GAGCTGCCCTCGGAGGCCGG and antisense CTCATTATTCCCCCGGCTTG) and subjected to melting curve analysis. As an internal control, GAPDH was amplified. All RT-PCR tests were performed in triplicate. The data were analyzed using the comparative Ct method.

### Western blot analysis

24 h after siRNA transfection, whole cell proteins were extracted by protein extraction reagents kit (Pierce, USA) and quantified using BCA kit (Thermo Scientific, USA). Equal amounts of protein (20 μg per lane) were electrophoresed under non-reducing conditions on 10% acrylamide gels. After SDS-PAGE, proteins were transferred to polyvinylidene difluoride membranes. Blots were blocked with 5% nonfat skim milk in tris-buffered saline/0.1% tween-20 and incubated overnight with primary antibody against p73 (1:300, Abcam, USA) at 4°C, and then with the horseradish peroxidase-conjugated secondary antibody (1:5000) for 2 h. Bands were normalized to GAPDH expression which was used as an internal control. Results from at least 3 separate experiments were analyzed.

The Huh-7 and Hep3B cells received or not received p73 siRNA pretreatment were seeded at a density of 1 × 10^5^ cells/well in 6-well plates and treated with NT4-37AA/rAAV or empty virus or nothing, respectively, at a multiplicity of infection (MOI) of 100. 72 hours after infection, western blot was performed with the same technique mentioned above.

Whole xenograft tumor tissues proteins were extracted by protein extraction reagents kit (Pierce, USA) and quantified using BCA kit (Thermo Scientific, USA). Western blotting was performed with the same technique mentioned above. The following primary antibodies were used to detect the proteins: anti-p73 (1:300, Abcam, USA),anti-GAPDH (1:2000, ab181602, Abcam, Cambridge, UK).

### Co-immunoprecipitation (CO-IP)

The iASPP (Abcam, USA) and p73 (Abcam, USA) antibodies were used in the CO-IP assays. Total protein lysate was obtained in immunoprecipitation buffer. The total protein concentration of the supernatants was quantified using a Bio-Rad DC™ Protein Assay Reagent A/B/S (Bio-Rad, USA). 500 μg of total protein was mixed with 1 μg the primary antibody, or IgG, and the mixture were shaken on a rotating shaker at 4°C for 2 hours. Beads (Protein G Sepharose 4 Fast Flow, GE Healthcare Life Sciences, Piscataway, NJ, USA) were added to the mixture and shaken at 4°C for 1 hour. Then the beads were collected by centrifugation and washed three times by immunoprecipitation buffer. 2 × sample loading buffer was added to the beads before boiling for 5 minutes. The supernatant was collected and used in the CO-IP assays.

### Colony formation assay

For colony formation assay, 1 × 10^3^ HCC cells (Huh7 and Hep3B), receiving or not receiving p73 siRNA pretreatment, treated with NT4-37AA/rAAV or empty virus were plated onto 60 mm dishes and incubated for 2 weeks before staining with crystal violet. Colonies, composed by 20–25 cells, were quantified under phase-contrast light microscopy. All the experiments were performed in triplicates.

### Proliferation assay

The effect of NT4-37AA/rAAV on HCC cell proliferation was measured by a MTT [3-(4,5-dimethylthiazol-2-yl)-2,5-diphenyltetrazolium bromide, Sigma corporation, USA] colorimetric assay. The HCC cells received or not received p73 siRNA pretreatment were seeded into 96-well plates at a density of 1 × 10^4^ cells per well and treated with NT4-37AA/rAAV or empty virus, respectively, at a MOI of 100. Then the cells were incubated in RPMI-1640 medium for 24, 48, and 72 h after treatment, respectively. 20 ml of MTT (5 mg/ml) was added into each well and the cell culture was continued for 4 h. Then the cells were lysed with DMSO (Sigma corporation, USA) and the absorbance value (OD) was measured at a wave length of 490 nm using a microplate reader.

### Flow cytometry

An Annexin-V-FLUOS Staining Kit (Roche Diagnostics, Indianapolis, IN, USA) was used to analyze the level of apoptosis. Procedures were done according to the manufacturer's guidelines. Briefly, 1 × 10^6^ cells were washed with PBS, centrifuged at 1000r/min for 5 min and then suspended in 100 μL of Annexin-V-Fluos staining solution. Flow cytometry analysis was performed with a FACScan flow cytometer (BD Biosciences, San Jose, CA, USA). Data were collected and analyzed with MODFIT software (BD Biosciences).

### Immunohistochemistry (IHC)

The sections were deparaffinized and rehydrated. Antigen retrieval in citrate buffer, and endogenous peroxidase activity was blocked using 0.3% hydrogen peroxide for 15 min. The sections were blocked for 30 min using 10% goat plasma. After incubation with primary antibody directed against Ki-67 (9027; Cell Signaling Technology, Danvers, MA, USA) for 1 h at 37°C, the cells were incubated with an HRP-conjugated secondary antibody (Goldenbridge Biotechnology, Zhongshan, China) according to the manufacturer's recommendations. Finally, the sections were visualized with diaminobenzidine and counterstained with hematoxylin, then dehydrated in alcohol and xylene and mounted onto glass slides. The number of Ki67-positive proliferation hepatocytes were counted from 6 randomly selected fields of each sample in the operative region under a magnification of × 400.

### Mouse xenograft HCC model

Male nude BALB/c mice weighing between 18 and 22 g 6 weeks old were purchased from the Laboratory Animal Center of Xi’an Jiaotong University Health Science Center (Xi’an, China), and housed under standard specific pathogen free conditions with access to sterilized water and food ad libitum. All of the animal experimental procedures were performed in accordance with the “Guide for the care and use of laboratory animals” (NIH publication No. 85–23, revised in 1996). All procedures were reviewed and approved by the Ethics Committee, Xi’an Jiaotong University Health Science Center.

72 mice were randomly divided into four groups, with 18 animals in each group. Before xenografting, the Huh-7 cells receiving or not receiving p73 siRNA pretreatment were treated with NT4-37AA/rAAV or empty virus, respectively, at a MOI of 100. 72 hours after infection, cells in each group were collected and 1 × 10^6^ cells were suspended in 0.2 mL of serum-free DMEM with 50% Matrigel (BD Biosciences, USA) and subcutaneously injected into the dorsal flanks of nude mice. Tumor growth was monitored for 7, 14 and 21 days after inoculation and then all mice were euthanized and the tumor volume was estimated using the following formula: tumor volume = length × width × width/2 [27]. The xenograft tumor tissues were explanted for western blot and pathological examination.

### Data and statistical analysis

All values are expressed as the mean ± standard deviation. Statistical analysis was performed using SPSS 16.0 software (SPSS, Inc., Chicago, IL, USA). Differences among the groups were assessed using one-way analysis of variance. A *p* value of less than 0.05 was considered a statistically significant difference.
